# Bis{6-[4-(2-ethyoxyphenyl­diazen­yl)phen­oxy]hexa­nol} monohydrate

**DOI:** 10.1107/S1600536808043924

**Published:** 2009-02-11

**Authors:** Ran-zhe Lu, Min Zhang, Lu-na Han, Bin Wang, Hai-bo Wang

**Affiliations:** a College of Science, Nanjing University of Technology, Xinmofan Road No. 5 Nanjing, Nanjing 210009, People’s Republic of China

## Abstract

The asymmetric unit of the title compound, 2C_20_H_26_N_2_O_3_·H_2_O, contains two independent mol­ecules and one water mol­ecule. The azo bonds adopt *trans* conformations and the dihedral angles between the aromatic rings in the two organic mol­ecules are 4.5 (2) and 1.5 (2)°. In the crystal structure, O—H⋯O and C—H⋯O hydrogen bonds help to establish the packing.

## Related literature

For the synthesis, see: Zhao *et al.* (2002 ▶). For background, see: Bach *et al.* (1996 ▶).
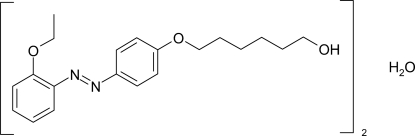


## Experimental

### 

#### Crystal data

2C_20_H_26_N_2_O_3_·H_2_O
*M* *_r_* = 702.87
Triclinic, 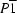

*a* = 7.4586 (7) Å
*b* = 11.5674 (11) Å
*c* = 24.070 (2) Å
α = 90.46 (3)°
β = 98.46 (3)°
γ = 106.10 (3)°
*V* = 1970.9 (5) Å^3^
*Z* = 2
Mo *K*α radiation
μ = 0.08 mm^−1^
*T* = 293 (2) K
0.30 × 0.20 × 0.10 mm

#### Data collection

Enraf–Nonius CAD-4 diffractometer
Absorption correction: ψ scan (North *et al.*, 1968 ▶) *T*_min_ = 0.956, *T*_max_ = 0.982
7726 measured reflections
7134 independent reflections
3403 reflections with *I* > 2σ(*I*)
*R*_int_ = 0.077
3 standard reflections every 200 reflections intensity decay: 1%

#### Refinement

*R*[*F* ^2^ > 2σ(*F* ^2^)] = 0.074
*wR*(*F* ^2^) = 0.181
*S* = 1.00
7134 reflections
460 parameters
H-atom parameters constrained
Δρ_max_ = 0.21 e Å^−3^
Δρ_min_ = −0.30 e Å^−3^

### 

Data collection: *CAD-4 EXPRESS* (Enraf–Nonius, 1994 ▶); cell refinement: *CAD-4 EXPRESS*; data reduction: *XCAD4* (Harms & Wocadlo, 1995 ▶); program(s) used to solve structure: *SHELXS97* (Sheldrick, 2008 ▶); program(s) used to refine structure: *SHELXL97* (Sheldrick, 2008 ▶); molecular graphics: *SHELXTL* (Sheldrick, 2008 ▶); software used to prepare material for publication: *SHELXL97*.

## Supplementary Material

Crystal structure: contains datablocks global, I. DOI: 10.1107/S1600536808043924/hb2877sup1.cif

Structure factors: contains datablocks I. DOI: 10.1107/S1600536808043924/hb2877Isup2.hkl

Additional supplementary materials:  crystallographic information; 3D view; checkCIF report

## Figures and Tables

**Table 1:** Hydrogen-bond geometry (Å, °)

*D*—H⋯*A*	*D*—H	H⋯*A*	*D*⋯*A*	*D*—H⋯*A*
O*W*—H*WB*⋯O4	0.85	2.32	2.713 (4)	109
O*W*—H*WA*⋯O4	0.85	2.31	2.713 (4)	110
O1—H1*A*⋯O*W*^i^	0.85	2.14	2.768 (4)	130
O4—H4*C*⋯O1^ii^	0.85	2.16	2.753 (4)	127
C15—H15*A*⋯O2^iii^	0.93	2.59	3.494 (5)	166
C35—H35*A*⋯O5^iii^	0.93	2.57	3.487 (5)	170
C40—H40*C*⋯O*W*^iv^	0.96	2.59	3.332 (5)	134

Symmetry codes: (i) 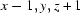; (ii) 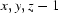; (iii) 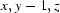; (iv) 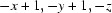.

**Fig. 1.**
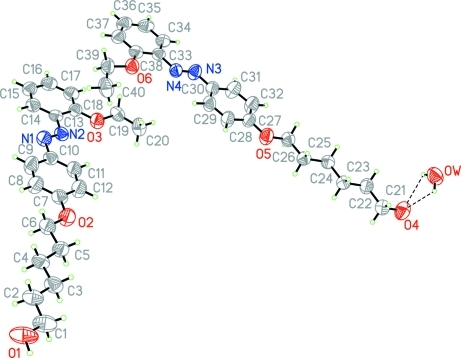
A view of the molecular structure of (I), showing displacement ellipsoids at the 30% probability level. Dashed lines indicate hydrogen bonds.

## supplementary crystallographic information

### Comment

The photophysical properties of azo compounds are of interest in the development of nonlinear optical and optical data storage materials (e.g. Bach *et al.*, 1996). As part of our studies in this area, we report herein the synthesis and crystal structure of the title compound, (I), (Fig. 1).

The title compound, C_20_H_26_N_2_O_3_, contains two independent molecules and one water molecule, which form intermolecular O—H..O and C—H···O hydrogen bonds (Table 1).

### Experimental

The title compound was prepared by the literature method (Zhao *et al.*, 2002). Yellow blocks of (I) were obtained by slow evaporation of an ethanol solution.

### Refinement

The H were placed geometrically with C—H = 0.93–0.97 Å and O—H = 0.85Å and refined as riding with *U*_iso_(H) = 1.2U_eq_(C,O) or 1.5*U*_eq_(methyl C).

### Crystal data

**Table:**

2C_20_H_26_N_2_O_3_·H_2_O	*Z* = 2
*M**_r_* = 702.87	*F*(000) = 756
Triclinic, *P*1	*D*_x_ = 1.184 Mg m^−^^3^
Hall symbol: -P 1	Mo *K*α radiation, λ = 0.71073 Å
*a* = 7.4586 (7) Å	Cell parameters from 25 reflections
*b* = 11.5674 (11) Å	θ = 9–13°
*c* = 24.070 (2) Å	µ = 0.08 mm^−^^1^
α = 90.46 (3)°	*T* = 293 K
β = 98.46 (3)°	Block, yellow
γ = 106.10 (3)°	0.30 × 0.20 × 0.10 mm
*V* = 1970.9 (5) Å^3^	

### Data collection

**Table:**

Enraf–Nonius CAD-4 diffractometer	3403 reflections with *I* > 2σ(*I*)
Radiation source: fine-focus sealed tube	*R*_int_ = 0.077
graphite	θ_max_ = 25.3°, θ_min_ = 1.7°
ω/2θ scans	*h* = 0→8
Absorption correction: ψ scan (North *et al.*, 1968)	*k* = −13→13
*T*_min_ = 0.956, *T*_max_ = 0.982	*l* = −28→28
7726 measured reflections	3 standard reflections every 200 reflections
7134 independent reflections	intensity decay: 1%

### Refinement

**Table:**

Refinement on *F*^2^	Primary atom site location: structure-invariant direct methods
Least-squares matrix: full	Secondary atom site location: difference Fourier map
*R*[*F*^2^ > 2σ(*F*^2^)] = 0.074	Hydrogen site location: inferred from neighbouring sites
*wR*(*F*^2^) = 0.181	H-atom parameters constrained
*S* = 1.00	*w* = 1/[σ^2^(*F*_o_^2^) + (0.05*P*)^2^ + 1.3*P*] where *P* = (*F*_o_^2^ + 2*F*_c_^2^)/3
7134 reflections	(Δ/σ)_max_ < 0.001
460 parameters	Δρ_max_ = 0.21 e Å^−^^3^
0 restraints	Δρ_min_ = −0.30 e Å^−^^3^

### Special details

**Table:**

Geometry. All e.s.d.'s (except the e.s.d. in the dihedral angle between two l.s. planes) are estimated using the full covariance matrix. The cell e.s.d.'s are taken into account individually in the estimation of e.s.d.'s in distances, angles and torsion angles; correlations between e.s.d.'s in cell parameters are only used when they are defined by crystal symmetry. An approximate (isotropic) treatment of cell e.s.d.'s is used for estimating e.s.d.'s involving l.s. planes.
Refinement. Refinement of *F*^2^ against ALL reflections. The weighted *R*-factor *wR* and goodness of fit *S* are based on *F*^2^, conventional *R*-factors *R* are based on *F*, with *F* set to zero for negative *F*^2^. The threshold expression of *F*^2^ > σ(*F*^2^) is used only for calculating *R*-factors(gt) *etc*. and is not relevant to the choice of reflections for refinement. *R*-factors based on *F*^2^ are statistically about twice as large as those based on *F*, and *R*- factors based on ALL data will be even larger.

### Fractional atomic coordinates and isotropic or equivalent isotropic displacement parameters (Å^2^)

**Table:**

	*x*	*y*	*z*	*U*_iso_*/*U*_eq_	
OW	0.5276 (4)	0.9282 (2)	−0.24533 (12)	0.0971 (10)	
HWB	0.4522	0.8576	−0.2505	0.117*	
HWA	0.4667	0.9734	−0.2339	0.117*	
O1	−0.2094 (4)	0.8215 (3)	0.72415 (13)	0.1066 (11)	
H1A	−0.2390	0.8873	0.7249	0.128*	
O2	−0.2623 (4)	0.2599 (2)	0.47771 (11)	0.0815 (8)	
O3	−0.2369 (4)	−0.2649 (2)	0.26105 (10)	0.0694 (7)	
N1	−0.2457 (5)	−0.2022 (3)	0.41759 (13)	0.0670 (9)	
N2	−0.2520 (4)	−0.2221 (2)	0.36679 (13)	0.0592 (8)	
C1	−0.2298 (6)	0.7803 (4)	0.66703 (19)	0.0878 (14)	
H1C	−0.1298	0.8320	0.6495	0.105*	
H1D	−0.3495	0.7864	0.6472	0.105*	
C2	−0.2224 (6)	0.6527 (3)	0.66147 (16)	0.0724 (11)	
H2B	−0.3242	0.6014	0.6784	0.087*	
H2C	−0.1043	0.6467	0.6825	0.087*	
C3	−0.2381 (6)	0.6057 (3)	0.60160 (16)	0.0725 (11)	
H3A	−0.1302	0.6523	0.5856	0.087*	
H3B	−0.3509	0.6176	0.5796	0.087*	
C4	−0.2468 (5)	0.4737 (3)	0.59679 (15)	0.0639 (10)	
H4A	−0.3572	0.4271	0.6117	0.077*	
H4B	−0.1363	0.4615	0.6200	0.077*	
C5	−0.2556 (6)	0.4259 (3)	0.53730 (15)	0.0674 (10)	
H5A	−0.3703	0.4327	0.5144	0.081*	
H5B	−0.1490	0.4747	0.5214	0.081*	
C6	−0.2523 (6)	0.2965 (3)	0.53547 (15)	0.0682 (11)	
H6A	−0.1369	0.2886	0.5575	0.082*	
H6B	−0.3589	0.2464	0.5509	0.082*	
C7	−0.2574 (5)	0.1447 (3)	0.46608 (16)	0.0641 (10)	
C8	−0.2501 (6)	0.0598 (3)	0.50434 (16)	0.0780 (12)	
H8A	−0.2476	0.0779	0.5422	0.094*	
C9	−0.2466 (6)	−0.0536 (4)	0.48678 (16)	0.0810 (13)	
H9A	−0.2415	−0.1114	0.5131	0.097*	
C10	−0.2504 (5)	−0.0821 (3)	0.43104 (15)	0.0578 (9)	
C11	−0.2537 (6)	0.0043 (3)	0.39299 (16)	0.0712 (11)	
H11A	−0.2522	−0.0129	0.3553	0.085*	
C12	−0.2592 (6)	0.1153 (4)	0.40993 (16)	0.0804 (13)	
H12A	−0.2643	0.1728	0.3834	0.096*	
C13	−0.2518 (5)	−0.3409 (3)	0.35068 (15)	0.0527 (9)	
C14	−0.2609 (5)	−0.4337 (3)	0.38626 (16)	0.0648 (10)	
H14A	−0.2637	−0.4199	0.4242	0.078*	
C15	−0.2659 (5)	−0.5478 (3)	0.36662 (18)	0.0702 (11)	
H15A	−0.2720	−0.6102	0.3910	0.084*	
C16	−0.2618 (5)	−0.5669 (4)	0.31014 (19)	0.0725 (11)	
H16A	−0.2657	−0.6431	0.2965	0.087*	
C17	−0.2520 (5)	−0.4757 (3)	0.27399 (17)	0.0656 (10)	
H17A	−0.2481	−0.4901	0.2362	0.079*	
C18	−0.2479 (5)	−0.3613 (3)	0.29358 (15)	0.0556 (9)	
C19	−0.2130 (6)	−0.2753 (3)	0.20418 (15)	0.0675 (11)	
H19A	−0.3237	−0.3317	0.1829	0.081*	
H19B	−0.1040	−0.3042	0.2016	0.081*	
C20	−0.1848 (6)	−0.1522 (4)	0.18134 (17)	0.0864 (13)	
H20A	−0.1668	−0.1557	0.1428	0.130*	
H20B	−0.0755	−0.0972	0.2029	0.130*	
H20C	−0.2940	−0.1250	0.1838	0.130*	
O4	0.1625 (4)	0.8614 (2)	−0.22791 (12)	0.0906 (9)	
H4C	0.0704	0.8070	−0.2458	0.109*	
O5	0.2453 (4)	0.3308 (2)	0.01659 (10)	0.0768 (8)	
O6	0.2785 (4)	−0.1956 (2)	0.24180 (10)	0.0668 (7)	
N3	0.2699 (5)	−0.1229 (3)	0.08391 (13)	0.0689 (9)	
N4	0.2798 (4)	−0.1396 (2)	0.13469 (12)	0.0581 (8)	
C21	0.1799 (6)	0.8282 (4)	−0.17127 (19)	0.0852 (13)	
H21A	0.0667	0.8301	−0.1564	0.102*	
H21B	0.2857	0.8874	−0.1494	0.102*	
C22	0.2088 (6)	0.7064 (3)	−0.16406 (16)	0.0707 (11)	
H22A	0.3261	0.7065	−0.1769	0.085*	
H22B	0.1073	0.6484	−0.1881	0.085*	
C23	0.2158 (6)	0.6645 (3)	−0.10461 (16)	0.0661 (10)	
H23A	0.3228	0.7186	−0.0808	0.079*	
H23B	0.1020	0.6683	−0.0906	0.079*	
C24	0.2325 (6)	0.5374 (3)	−0.10095 (15)	0.0658 (10)	
H24A	0.1279	0.4842	−0.1260	0.079*	
H24B	0.3484	0.5346	−0.1139	0.079*	
C25	0.2330 (6)	0.4913 (3)	−0.04241 (15)	0.0664 (10)	
H25A	0.3431	0.5407	−0.0179	0.080*	
H25B	0.1217	0.4995	−0.0282	0.080*	
C26	0.2352 (6)	0.3623 (3)	−0.04032 (15)	0.0671 (10)	
H26A	0.1215	0.3109	−0.0626	0.081*	
H26B	0.3435	0.3519	−0.0555	0.081*	
C27	0.2510 (5)	0.2175 (3)	0.03082 (15)	0.0614 (10)	
C28	0.2504 (6)	0.1940 (3)	0.08671 (16)	0.0737 (12)	
H28A	0.2452	0.2539	0.1119	0.088*	
C29	0.2573 (6)	0.0849 (3)	0.10573 (15)	0.0677 (11)	
H29A	0.2566	0.0707	0.1437	0.081*	
C30	0.2656 (5)	−0.0058 (3)	0.06852 (15)	0.0616 (10)	
C31	0.2649 (7)	0.0181 (4)	0.01333 (16)	0.0855 (14)	
H31A	0.2687	−0.0422	−0.0119	0.103*	
C32	0.2589 (7)	0.1291 (4)	−0.00667 (17)	0.0846 (13)	
H32A	0.2601	0.1436	−0.0446	0.102*	
C33	0.2824 (5)	−0.2583 (3)	0.14889 (15)	0.0536 (9)	
C34	0.2858 (5)	−0.3467 (3)	0.11001 (16)	0.0619 (10)	
H34A	0.2862	−0.3292	0.0724	0.074*	
C35	0.2887 (5)	−0.4604 (3)	0.12658 (19)	0.0705 (11)	
H35A	0.2898	−0.5191	0.1001	0.085*	
C36	0.2898 (5)	−0.4861 (3)	0.1814 (2)	0.0741 (11)	
H36A	0.2926	−0.5627	0.1922	0.089*	
C37	0.2870 (5)	−0.4001 (3)	0.22214 (17)	0.0679 (11)	
H37A	0.2871	−0.4187	0.2597	0.081*	
C38	0.2839 (5)	−0.2860 (3)	0.20512 (16)	0.0580 (9)	
C39	0.2613 (6)	−0.2236 (4)	0.29842 (15)	0.0755 (12)	
H39A	0.3771	−0.2374	0.3173	0.091*	
H39B	0.1586	−0.2960	0.2996	0.091*	
C40	0.2227 (7)	−0.1189 (4)	0.32730 (17)	0.0897 (14)	
H40A	0.2108	−0.1361	0.3658	0.135*	
H40B	0.1075	−0.1062	0.3085	0.135*	
H40C	0.3252	−0.0477	0.3261	0.135*	

### Atomic displacement parameters (Å^2^)

**Table:**

	*U*^11^	*U*^22^	*U*^33^	*U*^12^	*U*^13^	*U*^23^
OW	0.102 (2)	0.0739 (19)	0.120 (2)	0.0148 (17)	0.0522 (19)	−0.0047 (17)
O1	0.111 (3)	0.095 (2)	0.123 (3)	0.0508 (19)	0.008 (2)	−0.050 (2)
O2	0.128 (3)	0.0624 (17)	0.0648 (18)	0.0391 (17)	0.0244 (16)	−0.0029 (13)
O3	0.097 (2)	0.0609 (16)	0.0555 (16)	0.0248 (15)	0.0232 (14)	−0.0032 (13)
N1	0.089 (2)	0.062 (2)	0.055 (2)	0.0276 (18)	0.0165 (17)	−0.0053 (15)
N2	0.066 (2)	0.0560 (19)	0.059 (2)	0.0185 (15)	0.0193 (16)	−0.0019 (15)
C1	0.091 (3)	0.073 (3)	0.102 (4)	0.031 (3)	0.010 (3)	−0.029 (3)
C2	0.065 (3)	0.073 (3)	0.081 (3)	0.026 (2)	0.006 (2)	−0.019 (2)
C3	0.083 (3)	0.057 (2)	0.081 (3)	0.021 (2)	0.025 (2)	−0.011 (2)
C4	0.065 (3)	0.060 (2)	0.072 (3)	0.0217 (19)	0.018 (2)	−0.0085 (19)
C5	0.083 (3)	0.061 (2)	0.065 (2)	0.029 (2)	0.015 (2)	−0.0009 (19)
C6	0.087 (3)	0.066 (3)	0.055 (2)	0.024 (2)	0.015 (2)	−0.0097 (19)
C7	0.078 (3)	0.052 (2)	0.067 (3)	0.022 (2)	0.020 (2)	−0.008 (2)
C8	0.125 (4)	0.066 (3)	0.052 (2)	0.040 (3)	0.017 (2)	−0.002 (2)
C9	0.130 (4)	0.068 (3)	0.053 (2)	0.038 (3)	0.021 (2)	0.004 (2)
C10	0.063 (2)	0.050 (2)	0.063 (2)	0.0184 (18)	0.0158 (19)	−0.0029 (18)
C11	0.101 (3)	0.063 (3)	0.055 (2)	0.026 (2)	0.024 (2)	−0.004 (2)
C12	0.129 (4)	0.064 (3)	0.057 (3)	0.036 (3)	0.027 (2)	0.002 (2)
C13	0.048 (2)	0.050 (2)	0.063 (2)	0.0176 (17)	0.0127 (17)	−0.0029 (18)
C14	0.071 (3)	0.058 (2)	0.071 (3)	0.022 (2)	0.020 (2)	0.003 (2)
C15	0.071 (3)	0.057 (2)	0.086 (3)	0.022 (2)	0.017 (2)	0.011 (2)
C16	0.073 (3)	0.057 (3)	0.091 (3)	0.023 (2)	0.015 (2)	−0.005 (2)
C17	0.072 (3)	0.057 (2)	0.070 (3)	0.021 (2)	0.013 (2)	−0.010 (2)
C18	0.057 (2)	0.052 (2)	0.058 (2)	0.0153 (18)	0.0094 (18)	0.0027 (18)
C19	0.066 (3)	0.075 (3)	0.061 (2)	0.016 (2)	0.018 (2)	−0.006 (2)
C20	0.100 (4)	0.084 (3)	0.073 (3)	0.015 (3)	0.026 (3)	0.009 (2)
O4	0.089 (2)	0.082 (2)	0.091 (2)	0.0152 (17)	−0.0022 (17)	0.0206 (17)
O5	0.114 (2)	0.0645 (17)	0.0610 (17)	0.0322 (16)	0.0270 (15)	0.0108 (13)
O6	0.0854 (19)	0.0619 (16)	0.0582 (16)	0.0240 (14)	0.0210 (13)	0.0053 (13)
N3	0.100 (3)	0.060 (2)	0.053 (2)	0.0308 (18)	0.0152 (18)	0.0057 (15)
N4	0.065 (2)	0.0559 (19)	0.057 (2)	0.0197 (16)	0.0174 (15)	0.0067 (15)
C21	0.090 (3)	0.078 (3)	0.099 (4)	0.032 (3)	0.037 (3)	0.024 (3)
C22	0.081 (3)	0.062 (2)	0.070 (3)	0.019 (2)	0.019 (2)	0.012 (2)
C23	0.071 (3)	0.059 (2)	0.075 (3)	0.023 (2)	0.020 (2)	0.0070 (19)
C24	0.075 (3)	0.063 (2)	0.069 (3)	0.027 (2)	0.024 (2)	0.0096 (19)
C25	0.075 (3)	0.064 (2)	0.068 (3)	0.029 (2)	0.017 (2)	0.0091 (19)
C26	0.076 (3)	0.069 (3)	0.060 (2)	0.023 (2)	0.019 (2)	0.009 (2)
C27	0.074 (3)	0.055 (2)	0.059 (2)	0.023 (2)	0.013 (2)	0.0095 (19)
C28	0.115 (4)	0.057 (2)	0.060 (3)	0.032 (2)	0.031 (2)	0.007 (2)
C29	0.093 (3)	0.070 (3)	0.047 (2)	0.028 (2)	0.024 (2)	0.0092 (19)
C30	0.074 (3)	0.057 (2)	0.059 (2)	0.023 (2)	0.018 (2)	0.0036 (18)
C31	0.151 (4)	0.069 (3)	0.051 (2)	0.052 (3)	0.021 (3)	0.001 (2)
C32	0.139 (4)	0.072 (3)	0.055 (2)	0.043 (3)	0.029 (3)	0.005 (2)
C33	0.054 (2)	0.047 (2)	0.063 (2)	0.0159 (17)	0.0123 (18)	0.0005 (17)
C34	0.062 (3)	0.062 (2)	0.067 (2)	0.023 (2)	0.0120 (19)	−0.0024 (19)
C35	0.066 (3)	0.058 (3)	0.091 (3)	0.024 (2)	0.011 (2)	−0.009 (2)
C36	0.068 (3)	0.051 (2)	0.104 (3)	0.018 (2)	0.014 (2)	0.008 (2)
C37	0.065 (3)	0.060 (3)	0.080 (3)	0.020 (2)	0.012 (2)	0.011 (2)
C38	0.053 (2)	0.053 (2)	0.070 (3)	0.0146 (18)	0.0137 (19)	−0.0017 (19)
C39	0.084 (3)	0.086 (3)	0.056 (3)	0.022 (2)	0.015 (2)	0.015 (2)
C40	0.112 (4)	0.095 (3)	0.059 (3)	0.016 (3)	0.027 (2)	−0.003 (2)

### Geometric parameters (Å, °)

**Table:**

OW—HWB	0.8500	C20—H20C	0.9600
OW—HWA	0.8500	O4—C21	1.417 (4)
O1—C1	1.424 (5)	O4—H4C	0.8501
O1—H1A	0.8499	O5—C27	1.368 (4)
O2—C7	1.371 (4)	O5—C26	1.417 (4)
O2—C6	1.436 (4)	O6—C38	1.376 (4)
O3—C18	1.359 (4)	O6—C39	1.419 (4)
O3—C19	1.415 (4)	N3—N4	1.234 (4)
N1—N2	1.234 (4)	N3—C30	1.414 (4)
N1—C10	1.434 (4)	N4—C33	1.422 (4)
N2—C13	1.425 (4)	C21—C22	1.492 (5)
C1—C2	1.497 (5)	C21—H21A	0.9700
C1—H1C	0.9700	C21—H21B	0.9700
C1—H1D	0.9700	C22—C23	1.513 (5)
C2—C3	1.513 (5)	C22—H22A	0.9700
C2—H2B	0.9700	C22—H22B	0.9700
C2—H2C	0.9700	C23—C24	1.512 (4)
C3—C4	1.513 (5)	C23—H23A	0.9700
C3—H3A	0.9700	C23—H23B	0.9700
C3—H3B	0.9700	C24—C25	1.511 (5)
C4—C5	1.516 (5)	C24—H24A	0.9700
C4—H4A	0.9700	C24—H24B	0.9700
C4—H4B	0.9700	C25—C26	1.499 (5)
C5—C6	1.504 (5)	C25—H25A	0.9700
C5—H5A	0.9700	C25—H25B	0.9700
C5—H5B	0.9700	C26—H26A	0.9700
C6—H6A	0.9700	C26—H26B	0.9700
C6—H6B	0.9700	C27—C28	1.375 (5)
C7—C8	1.358 (5)	C27—C32	1.378 (5)
C7—C12	1.388 (5)	C28—C29	1.357 (5)
C8—C9	1.383 (5)	C28—H28A	0.9300
C8—H8A	0.9300	C29—C30	1.394 (5)
C9—C10	1.373 (5)	C29—H29A	0.9300
C9—H9A	0.9300	C30—C31	1.359 (5)
C10—C11	1.364 (5)	C31—C32	1.384 (5)
C11—C12	1.357 (5)	C31—H31A	0.9300
C11—H11A	0.9300	C32—H32A	0.9300
C12—H12A	0.9300	C33—C34	1.389 (4)
C13—C14	1.372 (5)	C33—C38	1.393 (5)
C13—C18	1.399 (5)	C34—C35	1.382 (5)
C14—C15	1.387 (5)	C34—H34A	0.9300
C14—H14A	0.9300	C35—C36	1.355 (5)
C15—C16	1.382 (5)	C35—H35A	0.9300
C15—H15A	0.9300	C36—C37	1.397 (5)
C16—C17	1.365 (5)	C36—H36A	0.9300
C16—H16A	0.9300	C37—C38	1.390 (5)
C17—C18	1.392 (5)	C37—H37A	0.9300
C17—H17A	0.9300	C39—C40	1.507 (5)
C19—C20	1.501 (5)	C39—H39A	0.9700
C19—H19A	0.9700	C39—H39B	0.9700
C19—H19B	0.9700	C40—H40A	0.9600
C20—H20A	0.9600	C40—H40B	0.9600
C20—H20B	0.9600	C40—H40C	0.9600
			
HWB—OW—HWA	106.9	C21—O4—H4C	106.1
C1—O1—H1A	108.6	C27—O5—C26	120.5 (3)
C7—O2—C6	117.8 (3)	C38—O6—C39	118.1 (3)
C18—O3—C19	119.5 (3)	N4—N3—C30	115.4 (3)
N2—N1—C10	112.9 (3)	N3—N4—C33	114.2 (3)
N1—N2—C13	115.5 (3)	O4—C21—C22	113.7 (4)
O1—C1—C2	112.5 (4)	O4—C21—H21A	108.8
O1—C1—H1C	109.1	C22—C21—H21A	108.8
C2—C1—H1C	109.1	O4—C21—H21B	108.8
O1—C1—H1D	109.1	C22—C21—H21B	108.8
C2—C1—H1D	109.1	H21A—C21—H21B	107.7
H1C—C1—H1D	107.8	C21—C22—C23	115.2 (3)
C1—C2—C3	114.5 (3)	C21—C22—H22A	108.5
C1—C2—H2B	108.6	C23—C22—H22A	108.5
C3—C2—H2B	108.6	C21—C22—H22B	108.5
C1—C2—H2C	108.6	C23—C22—H22B	108.5
C3—C2—H2C	108.6	H22A—C22—H22B	107.5
H2B—C2—H2C	107.6	C24—C23—C22	112.4 (3)
C2—C3—C4	113.5 (3)	C24—C23—H23A	109.1
C2—C3—H3A	108.9	C22—C23—H23A	109.1
C4—C3—H3A	108.9	C24—C23—H23B	109.1
C2—C3—H3B	108.9	C22—C23—H23B	109.1
C4—C3—H3B	108.9	H23A—C23—H23B	107.9
H3A—C3—H3B	107.7	C25—C24—C23	113.7 (3)
C3—C4—C5	114.3 (3)	C25—C24—H24A	108.8
C3—C4—H4A	108.7	C23—C24—H24A	108.8
C5—C4—H4A	108.7	C25—C24—H24B	108.8
C3—C4—H4B	108.7	C23—C24—H24B	108.8
C5—C4—H4B	108.7	H24A—C24—H24B	107.7
H4A—C4—H4B	107.6	C26—C25—C24	113.0 (3)
C6—C5—C4	111.9 (3)	C26—C25—H25A	109.0
C6—C5—H5A	109.2	C24—C25—H25A	109.0
C4—C5—H5A	109.2	C26—C25—H25B	109.0
C6—C5—H5B	109.2	C24—C25—H25B	109.0
C4—C5—H5B	109.2	H25A—C25—H25B	107.8
H5A—C5—H5B	107.9	O5—C26—C25	108.1 (3)
O2—C6—C5	107.6 (3)	O5—C26—H26A	110.1
O2—C6—H6A	110.2	C25—C26—H26A	110.1
C5—C6—H6A	110.2	O5—C26—H26B	110.1
O2—C6—H6B	110.2	C25—C26—H26B	110.1
C5—C6—H6B	110.2	H26A—C26—H26B	108.4
H6A—C6—H6B	108.5	O5—C27—C28	116.0 (3)
C8—C7—O2	125.7 (3)	O5—C27—C32	124.4 (3)
C8—C7—C12	118.7 (3)	C28—C27—C32	119.6 (3)
O2—C7—C12	115.6 (3)	C29—C28—C27	121.1 (4)
C7—C8—C9	119.8 (4)	C29—C28—H28A	119.4
C7—C8—H8A	120.1	C27—C28—H28A	119.4
C9—C8—H8A	120.1	C28—C29—C30	120.2 (3)
C10—C9—C8	121.0 (4)	C28—C29—H29A	119.9
C10—C9—H9A	119.5	C30—C29—H29A	119.9
C8—C9—H9A	119.5	C31—C30—C29	118.2 (3)
C11—C10—C9	118.9 (3)	C31—C30—N3	117.2 (3)
C11—C10—N1	124.8 (3)	C29—C30—N3	124.6 (3)
C9—C10—N1	116.3 (3)	C30—C31—C32	122.3 (4)
C12—C11—C10	120.3 (4)	C30—C31—H31A	118.8
C12—C11—H11A	119.9	C32—C31—H31A	118.8
C10—C11—H11A	119.9	C27—C32—C31	118.5 (4)
C11—C12—C7	121.3 (4)	C27—C32—H32A	120.7
C11—C12—H12A	119.4	C31—C32—H32A	120.7
C7—C12—H12A	119.4	C34—C33—C38	118.6 (3)
C14—C13—C18	119.6 (3)	C34—C33—N4	123.6 (3)
C14—C13—N2	124.9 (3)	C38—C33—N4	117.7 (3)
C18—C13—N2	115.4 (3)	C35—C34—C33	120.8 (4)
C13—C14—C15	121.1 (4)	C35—C34—H34A	119.6
C13—C14—H14A	119.4	C33—C34—H34A	119.6
C15—C14—H14A	119.4	C36—C35—C34	119.9 (4)
C16—C15—C14	118.7 (4)	C36—C35—H35A	120.1
C16—C15—H15A	120.6	C34—C35—H35A	120.1
C14—C15—H15A	120.6	C35—C36—C37	121.5 (4)
C17—C16—C15	121.2 (4)	C35—C36—H36A	119.3
C17—C16—H16A	119.4	C37—C36—H36A	119.3
C15—C16—H16A	119.4	C38—C37—C36	118.3 (4)
C16—C17—C18	120.2 (4)	C38—C37—H37A	120.9
C16—C17—H17A	119.9	C36—C37—H37A	120.9
C18—C17—H17A	119.9	O6—C38—C37	122.8 (3)
O3—C18—C17	124.5 (3)	O6—C38—C33	116.3 (3)
O3—C18—C13	116.3 (3)	C37—C38—C33	120.9 (3)
C17—C18—C13	119.2 (3)	O6—C39—C40	108.2 (3)
O3—C19—C20	107.2 (3)	O6—C39—H39A	110.1
O3—C19—H19A	110.3	C40—C39—H39A	110.1
C20—C19—H19A	110.3	O6—C39—H39B	110.1
O3—C19—H19B	110.3	C40—C39—H39B	110.1
C20—C19—H19B	110.3	H39A—C39—H39B	108.4
H19A—C19—H19B	108.5	C39—C40—H40A	109.5
C19—C20—H20A	109.5	C39—C40—H40B	109.5
C19—C20—H20B	109.5	H40A—C40—H40B	109.5
H20A—C20—H20B	109.5	C39—C40—H40C	109.5
C19—C20—H20C	109.5	H40A—C40—H40C	109.5
H20A—C20—H20C	109.5	H40B—C40—H40C	109.5
H20B—C20—H20C	109.5		
			
C10—N1—N2—C13	−178.7 (3)	C30—N3—N4—C33	179.5 (3)
O1—C1—C2—C3	−178.4 (3)	O4—C21—C22—C23	−176.3 (3)
C1—C2—C3—C4	−175.2 (3)	C21—C22—C23—C24	176.3 (4)
C2—C3—C4—C5	−178.0 (3)	C22—C23—C24—C25	−178.0 (3)
C3—C4—C5—C6	176.6 (3)	C23—C24—C25—C26	175.7 (3)
C7—O2—C6—C5	178.7 (3)	C27—O5—C26—C25	−179.4 (3)
C4—C5—C6—O2	179.6 (3)	C24—C25—C26—O5	177.0 (3)
C6—O2—C7—C8	2.4 (6)	C26—O5—C27—C28	−176.6 (3)
C6—O2—C7—C12	−177.3 (4)	C26—O5—C27—C32	4.0 (6)
O2—C7—C8—C9	179.7 (4)	O5—C27—C28—C29	−179.4 (4)
C12—C7—C8—C9	−0.6 (7)	C32—C27—C28—C29	−0.1 (6)
C7—C8—C9—C10	0.0 (7)	C27—C28—C29—C30	0.1 (6)
C8—C9—C10—C11	1.4 (6)	C28—C29—C30—C31	−0.5 (6)
C8—C9—C10—N1	179.9 (4)	C28—C29—C30—N3	−178.9 (4)
N2—N1—C10—C11	−2.5 (5)	N4—N3—C30—C31	176.6 (4)
N2—N1—C10—C9	179.1 (4)	N4—N3—C30—C29	−5.1 (6)
C9—C10—C11—C12	−2.1 (6)	C29—C30—C31—C32	0.8 (7)
N1—C10—C11—C12	179.6 (4)	N3—C30—C31—C32	179.3 (4)
C10—C11—C12—C7	1.4 (7)	O5—C27—C32—C31	179.7 (4)
C8—C7—C12—C11	0.0 (7)	C28—C27—C32—C31	0.4 (6)
O2—C7—C12—C11	179.6 (4)	C30—C31—C32—C27	−0.8 (7)
N1—N2—C13—C14	5.6 (5)	N3—N4—C33—C34	4.0 (5)
N1—N2—C13—C18	−176.5 (3)	N3—N4—C33—C38	−176.9 (3)
C18—C13—C14—C15	0.1 (5)	C38—C33—C34—C35	0.7 (5)
N2—C13—C14—C15	177.9 (3)	N4—C33—C34—C35	179.8 (3)
C13—C14—C15—C16	0.0 (6)	C33—C34—C35—C36	−0.6 (6)
C14—C15—C16—C17	0.3 (6)	C34—C35—C36—C37	0.4 (6)
C15—C16—C17—C18	−0.6 (6)	C35—C36—C37—C38	−0.3 (6)
C19—O3—C18—C17	−5.3 (5)	C39—O6—C38—C37	−4.8 (5)
C19—O3—C18—C13	173.6 (3)	C39—O6—C38—C33	174.0 (3)
C16—C17—C18—O3	179.5 (3)	C36—C37—C38—O6	179.2 (3)
C16—C17—C18—C13	0.7 (5)	C36—C37—C38—C33	0.4 (5)
C14—C13—C18—O3	−179.4 (3)	C34—C33—C38—O6	−179.4 (3)
N2—C13—C18—O3	2.6 (4)	N4—C33—C38—O6	1.4 (5)
C14—C13—C18—C17	−0.4 (5)	C34—C33—C38—C37	−0.6 (5)
N2—C13—C18—C17	−178.5 (3)	N4—C33—C38—C37	−179.8 (3)
C18—O3—C19—C20	−173.9 (3)	C38—O6—C39—C40	−169.8 (3)

### Hydrogen-bond geometry (Å, °)

**Table:**

*D*—H···*A*	*D*—H	H···*A*	*D*···*A*	*D*—H···*A*
OW—HWB···O4	0.85	2.32	2.713 (4)	109
OW—HWA···O4	0.85	2.31	2.713 (4)	110
O1—H1A···OW^i^	0.85	2.14	2.768 (4)	130
O4—H4C···O1^ii^	0.85	2.16	2.753 (4)	127
C15—H15A···O2^iii^	0.93	2.59	3.494 (5)	166
C35—H35A···O5^iii^	0.93	2.57	3.487 (5)	170
C40—H40C···OW^iv^	0.96	2.59	3.332 (5)	134

Symmetry codes: (i) *x*−1, *y*, *z*+1; (ii) *x*, *y*, *z*−1; (iii) *x*, *y*−1, *z*; (iv) −*x*+1, −*y*+1, −*z*.

## References

[bb1] Bach, H., Anderle, K., Fuhrmann, Th. & Wendorff, J. H. (1996). *J. Phys. Chem.* **100**, 4135–4140.

[bb2] Enraf–Nonius (1994). *CAD-4 EXPRESS* Enraf–Nonius, Delft, The Netherlands.

[bb3] Harms, K. & Wocadlo, S. (1995). *XCAD4* University of Marburg, Germany.

[bb4] North, A. C. T., Phillips, D. C. & Mathews, F. S. (1968). *Acta Cryst.* A**24**, 351–359.

[bb5] Sheldrick, G. M. (2008). *Acta Cryst.* A**64**, 112–122. 10.1107/S0108767307043930 18156677

[bb6] Zhao, X. Y., Hu, X., Yue, C. Y., Xia, X. & Gan, L. H. (2002). *Thin Solid Films*, **417**, 95–100.

